# A Systematic Comparison of Alpha‐Synuclein Seed Amplification Assays for Increasing Reproducibility

**DOI:** 10.1002/acn3.70384

**Published:** 2026-04-01

**Authors:** Manuela Amaral‐do‐Nascimento, Daniela F. Santos, Tuane C. R. G. Vieira, Tiago F. Outeiro

**Affiliations:** ^1^ Instituto de Bioquímica Médica, Instituto Nacional de Ciência e Tecnologia de Biologia Estrutural e Bioimagem Universidade Federal do Rio de Janeiro Rio de Janeiro Brazil; ^2^ Faculdade de Medicina e Ciências Biomédicas, Algarve Biomedical Center Research Institute (ABC‐Ri), Algarve Biomedical Center (ABC) University of Algarve Faro Portugal; ^3^ Department of Experimental Neurodegeneration, Center for Biostructural Imaging of Neurodegeneration University Medical Center Göttingen Göttingen Germany; ^4^ Faculty of Medical Sciences, Translational and Clinical Research Institute Newcastle University Newcastle UK; ^5^ Max Planck Institute for Multidisciplinary Sciences Göttingen Germany; ^6^ Scientific Employee With an Honorary Contract at Deutsches Zentrum für Neurodegenerative Erkrankungen (DZNE) Göttingen Germany

**Keywords:** alpha‐synuclein, biomarker, RT‐QuIC, seed amplification assay, synucleinopathies

## Abstract

Seed amplification assays (SAAs) enable ultrasensitive detection of misfolded α‐synuclein across biofluids and tissues. Yet, heterogeneity in protocols limits cross‐study comparability and clinical translation. Here, we review α‐synuclein SAA methods and their performance across various biological matrices. We catalog key variables (e.g., human recombinant α‐synuclein variants, buffers, salts, beads, shaking, incubation temperature) and outline pre‐analytical factors that shape outcomes. We propose a prioritized framework of key analytical and pre‐analytical variables most likely to influence assay reproducibility and clinical interpretation. We also outline minimal reporting criteria and practical quality‐control checkpoints, and recommend multicenter interlaboratory proficiency testing as essential steps to accelerate the robust clinical and research implementation of α‐synuclein SAAs.

**Abbreviations**
μL: microliter
μM: micromolar
AD: Alzheimer's disease
aSyn: α‐synuclein
CBD: Corticobasal degeneration
CI: cognitively impaired
CJD: Creutzfeldt–Jakob disease
CU: cognitively unimpaired
DLB: dementia with Lewy bodies
ET: essential tremor
FCx: frontal cortex
FTD: frontotemporal dementia
FTLD‐TDP: Frontotemporal Lobar Degeneration with TDP‐43 pathology
HC: healthy controls
iAF: idiopathic autonomic failure
IDT: indeterminate parkinsonism disease
iRBD: idiopathic REM sleep behavior disorder
LB: Lewy body
LCOs: luminescent conjugated oligothiophenes
MCI: mild cognitive impairment
mg: milligram
mm: millimeters
mM: millimolar
MSA: multiple system atrophy
MSA‐C: Multiple system atrophy—cerebellar subtype
MSA‐P: Multiple system atrophy‐ parkinsonian type
ND: neurodegenerative disorders
nEVs: neuron‐derived extracellular vesicles
NMC: non‐manifesting carriers
NNC: non‐neurodegenerative control
OM: olfactory mucosa
PAF: pure autonomic failure
PB: phosphate buffer
PBS: phosphate‐buffered saline
PD: Parkinson's disease
PDD: Parkinson's disease dementia
PMCA: protein misfolding cyclic amplification assay
PrP: prion protein
PSP: progressive supranuclear palsy
RBD: REM sleep behavior disorder
RT‐QuIC: real‐time quaking‐induced conversion
SAA: Seed amplification assay
SNAP: Suspected Non‐Alzheimer's disease Pathophysiology
SNc: substantia nigra pars compacta
SOP: standard operating procedures
ThT: Thioflavin T
VE: viral encephalitis
VMAT2: vesicular monoamine transporter type 2

## 1 Introduction

Protein aggregation diseases are a broad and heterogeneous class of disorders characterized by the accumulation of misfolded, aggregated proteins that are thought to disrupt cellular homeostasis. These disorders span a broad spectrum, including systemic diseases such as type II diabetes, cancer, and neurodegenerative conditions, including Alzheimer's disease (AD), prion diseases, and Parkinson's disease (PD). Neurodegenerative conditions are actually classified by the type of protein aggregates accumulating in the central nervous system. The main protein components of the prototypical aggregates include amyloid‐β and tau in AD, prion protein (PrP) in prion diseases, and α‐synuclein (aSyn) in PD and related synucleinopathies [1].

A major challenge in managing these disorders arises due to the typically late onset of the characteristic clinical features, which appear only after substantial cellular damage has already occurred [2]. At those stages, opportunities for intervention are often lost. Therefore, the development of sensitive and specific methods capable of detecting molecular signatures of disease in early or even pre‐clinical stages has been a major focus of attention in the field of neurodegeneration.

A major breakthrough came with the discovery that misfolded proteins can act as seeds, catalyzing the conversion of their native counterparts into aggregated species [3]. This “templated misfolding” phenomenon, initially described in prion diseases, was later recognized in diseases associated with other proteins, including aSyn and tau proteins [4, 5]. These findings inspired the development of seed amplification assays (SAAs), biochemical techniques that exploit this self‐propagation property to detect trace amounts of pathogenic aggregates in biological samples.

Seed amplification assays (SAA) were initially pioneered in the prion field, where protein misfolding cyclic amplification assay (PMCA) and real‐time quaking‐induced conversion (RT‐QuIC) were developed to amplify and detect misfolded prion protein in biological samples. PMCA was first applied to prion detection, achieving attogram sensitivity in brain homogenates by relying on repeated cycles of sonication to fragment newly formed aggregates and thereby enable exponential amplification [6]. RT‐QuIC further expanded these capabilities by offering real‐time fluorescence monitoring via Thioflavin T (ThT) binding in high‐throughput plate formats [7], and using intermittent mechanical shaking to break down fibrils continuously. Building on these concepts, analogous assays were later adapted to non‐prion proteinopathies [8, 9], opening new avenues for diagnostics and research. In this review, we use the term aSyn‐SAA to refer to aSyn seed amplification platforms, while RT‐QuIC and PMCA are cited where appropriate to reflect the terminology used in specific original publications.

### 1.1 aSyn: From Key Player in Disease to Biomarker

aSyn, a 14 kDa presynaptic protein, plays vital roles in synaptic vesicle trafficking, dopamine regulation via vesicular monoamine transporter type 2 (VMAT2), mitochondrial function, and even epigenetic modulation [10, 11, 12, 13]. Under pathological conditions, aSyn undergoes a multistep process of misfolding and aggregation, transitioning from soluble monomers to oligomers and ultimately to amyloid fibrils [14]. These fibrils can further fragment and serve as seeds, propagating the pathology across cells and tissues.

This pathological aggregation defines a group of disorders known as synucleinopathies, which includes PD, dementia with Lewy bodies (DLB), multiple system atrophy (MSA), pure autonomic failure (PAF), and REM sleep behavior disorder (RBD) [2]. These diseases are characterized by the accumulation of intracellular inclusions composed of aggregated aSyn, among other protein and lipid components. The inclusions are known as Lewy bodies and Lewy neurites in PD and DLB (Lewy body diseases), and as glial cytoplasmic inclusions in MSA [15].

The detection of aSyn aggregates in biological fluids and tissues has become a central goal in biomarker discovery for synucleinopathies. Strikingly, the aSyn‐SAA demonstrated the capacity to detect aSyn seeds in cerebrospinal fluid (CSF), olfactory mucosa (OM), skin, and other peripheral tissues, making it one of the most promising tools for early diagnosis and disease classification [16, 17, 18]. Additionally, the assay holds promise for monitoring disease progression, distinguishing between aSyn strains, and guiding therapeutic development in clinical trials.

However, despite the growing usage of this assay in the field, there is still substantial variability in the aSyn‐SAA protocols used by the scientific community, including differences in substrate type, buffer composition, sample origin, incubation conditions, and fluorescence analysis. These variations impact assay performance, compromising inter‐study comparisons and hindering efforts to standardize the assay.

Here, we aim to provide a systematic, comprehensive, and critical comparison of SAA protocols applied to aSyn detection. We review technical parameters that influence assay sensitivity and specificity, including substrate selection, seed preparation, buffer systems, mechanical settings, and readout conditions. By summarizing the current state of the field, we seek to identify remaining sources of variability, highlight important points necessary for methodological consensus, and propose directions for future harmonization and clinical translation of aSyn‐SAA.

### 1.2 Principles of aSyn Seed Amplification and Clinical Readouts

**FIGURE 1:**
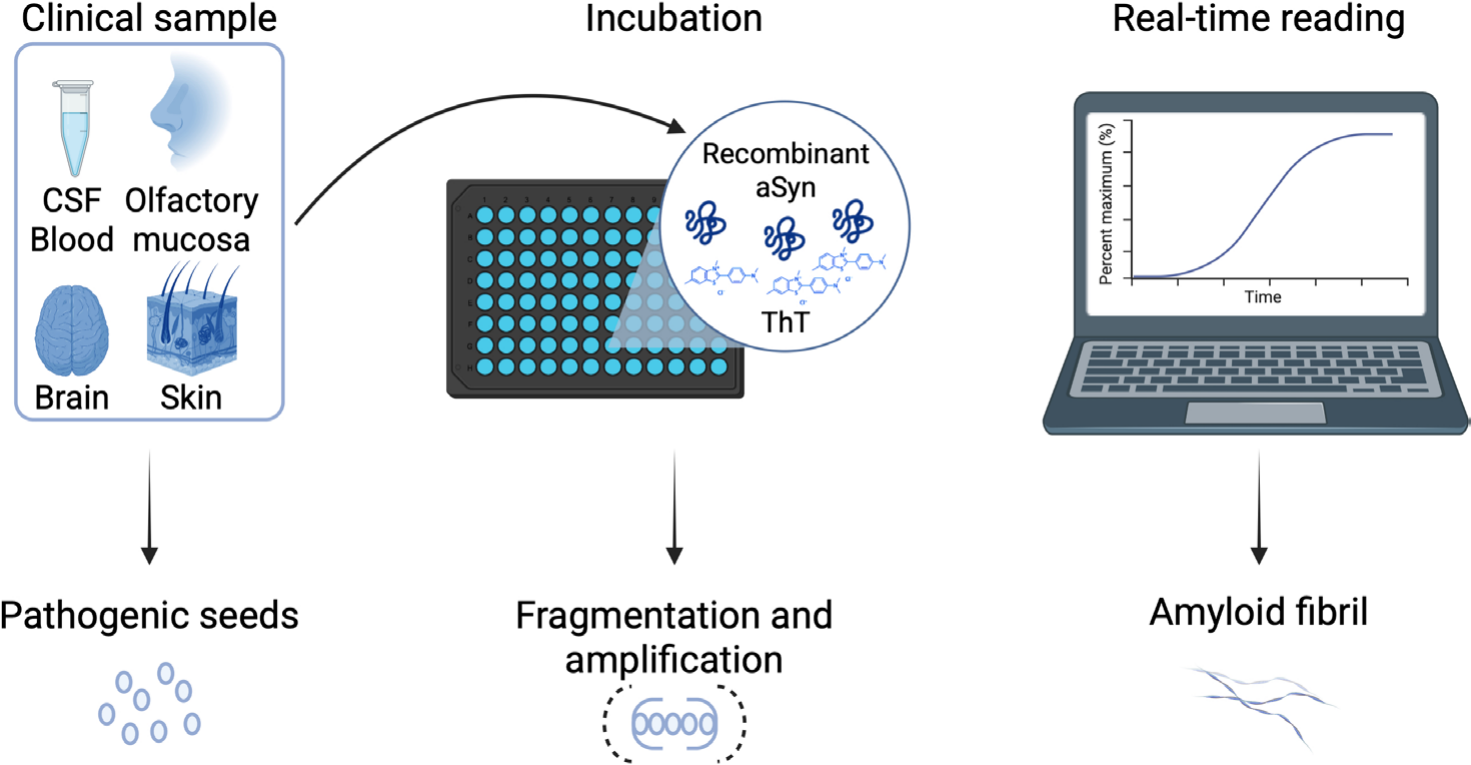
Principle of the seed amplification assay (SAA) applied to aSyn. Small amounts of pathological aggregates present in biological samples act as seeds to convert recombinant α‐synuclein into amyloid fibrils. The process is accelerated by cycles of agitation and incubation. Increased temperatures and the use of beads can enhance this process. Fibril formation is monitored in real time by fluorescence of the ThT dye. Created with https://BioRender.com.

As described above, the SAA is a highly sensitive and specific amplification method that was initially developed for detecting misfolded PrP and later adapted for other amyloidogenic proteins, including aSyn [19, 20, 21]. In this assay, pathological aSyn seeds present in biological samples act as templates to induce the misfolding and aggregation of recombinant aSyn substrate under intermittent shaking conditions. The kinetics of amyloid formation are monitored in real time by ThT fluorescence, which increases upon binding to β‐sheet‐rich fibrillar structures (Figure 1).

### 1.3 Seed‐Dependent Templated Misfolding

SAA exploits the nucleation‐dependent polymerization mechanism of amyloid formation. Small amounts of misfolded aSyn in a clinical sample initiate the aggregation of a soluble recombinant aSyn provided in the reaction mix. The process is accelerated through cycles of shaking and incubation, which facilitate seed–substrate interactions and fibril fragmentation, generating new seeding‐competent species. ThT fluorescence emission provides a kinetic readout, with shorter lag phases and higher fluorescence intensities indicating more vigorous seeding activity. The specificity of the assay is enhanced by optimizing parameters such as buffer composition, ionic strength, pH, temperature, and substrate sequence or conformation [20, 21].

## 2 Clinical and Translational Applications of aSyn‐SAA

The utility of the aSyn‐SAAs extends far beyond the simple detection of misfolded proteins. It has evolved into a versatile analytical tool that serves both clinical diagnostic objectives and experimental research goals, offering unique insights into the molecular pathology of synucleinopathies and the dynamics of protein aggregation.

In clinical contexts, the aSyn‐SAA has emerged as one of the most promising methods for the detection of aSyn pathology, providing a molecular biomarker for diseases previously diagnosed solely by clinical criteria or post‐mortem examination [2]. Several studies have demonstrated that aSyn‐SAA applied to CSF can achieve diagnostic sensitivities and specificities exceeding 90% for PD and DLB, distinguishing these conditions from healthy controls and from other neurodegenerative disorders (Table 1) [62].

**TABLE 1:** SAA conditions reported in aSyn studies.

Seed source	Sensitivity	Specificity	References
Brain	DLB FCx 70%, SNc 75%	95%	[22]
Brain	94%	100%	[23]
Brain	Not stated	Not stated	[24]
Brain	Not stated	Not stated	[25]
Brain	Not stated	Not stated	[26]
Brain homogenates	Qualitative	Qualitative	[27]
CSF	DLB 92%, DLB/ad 65%, PD 95%	100%	[28]
CSF	88.5%	96.9%	[29]
CSF	93%	100%	[8]
CSF	LRRK2‐PD: 40%, IPD: 90%, LRRK2‐NMC: 18.8%	IPD: 80%	[30]
CSF	98%–97.1%	98.4%	[31]
CSF	89%	Not stated	[32]
CSF	84%	93%	[33]
CSF	97.2%	87.1%	[34]
CSF	95.3%	98%	[35]
CSF	PD: 93.6%, MSA: 84.6%	100%	[36]
CSF	100% discovery, ~95% confirmatory	100% MSA versus controls	[37]
CSF	Not stated	Not stated	[38]
CSF	90%	90.4%	[39]
CSF	95.1%	86.7%–98.3%	[40]
CSF	AbbVie: 89%, Amprion: 96%, Caughey: 86%	AbbVie: 100%, Amprion/Caughey: 97%	[41]
CSF	PD: 75%–81%, MSA: 12%–9%	100% versus CS; 89%–91% vs. tauopathies	[42]
CSF	LBD vesus MSA and PSP: 95%	LBD versus MSA and PSP: 67%	[43]
CSF	Discovery: 91.9%, Validation: 80.7%	Discovery: 85.3%, Validation: 76.5%	[44]
CSF	PD: 89.2%, MSA: 75%, iRBD: 64.4%	96%	[45]
CSF	Not stated	Not stated	[46]
CSF	PD versus control: 78.8%, PD versus CBD: 71%, PD versus PSP: 75%	PD: 100%, MSA: 92.6%	[47]
CSF	PD: 87.5%, PD versus HC: 96.3%	Not stated	[48]
CSF	Not stated	Not stated	[49]
CSF	PD: 8%, iRBD: 93%	Not stated	[50]
CSF	DLB: 72%	Controls: 96%	[51]
CSF	LBD+: 88%, LBD‐: 17%	Not stated	[52]
CSF	low	low	[53]
CSF	Not stated	Not stated	[54]
CSF	92.9%	Not stated	[55]
CSF	Not stated	Not stated	[56]
CSF	Not stated	Not stated	[57]
CSF	95.8%	87%	[58]
CSF	Not stated	Not stated	[59]
CSF	UK PD: 96%, PSP: 15%; PPMI PD: 73%	Not stated	[60]
CSF	PD: 81.6%–91.8%, MSA: 92.1%	PSP: 90.0%–100%, HC: 87.0%–100%, MSA: 96.5%	[61]
CSF	96.2%	82.3%	[32]

*Note:* +SAA conditions reported in selected studies of synucleinopathies. Seed source types include brain regions or CSF. Sensitivities and specificities are expressed as percentages or described qualitatively when numerical values were not reported.

These results are paving the way for the use of aSyn‐SAA as a supportive diagnostic tool, particularly in early‐stage or atypical presentations where clinical signs may be ambiguous. Moreover, by adapting the assay to peripheral tissues and fluids, researchers are developing less invasive approaches that may facilitate wider screening, particularly in aging populations or high‐risk individuals.

**FIGURE 2:**
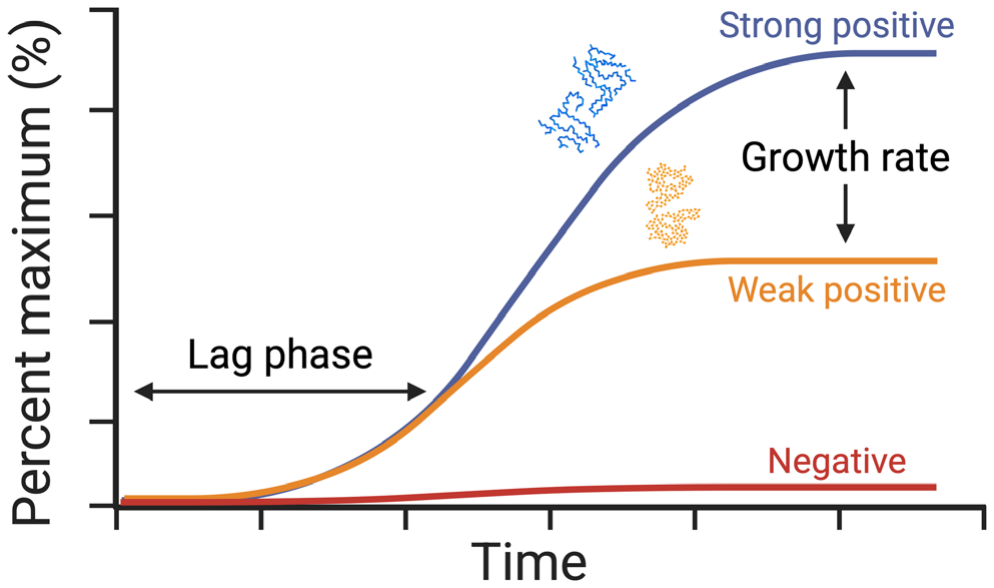
Representative SAA kinetic profiles for aSyn. ThT fluorescence is monitored in real time, allowing the identification of parameters such as induction time (lag phase), exponential growth rate, and maximum signal intensity (plateau). These parameters are used to assess the presence and intensity of seeding activity in clinical samples. Differences in seeding activity have been correlated to different aSyn strains, with PD showing a stronger ThT signal than MSA, for example. Created with https://BioRender.com.

In addition to its role in aiding diagnosis, aSyn‐SAA holds promise for disease monitoring and prognosis. Although still under investigation, kinetic parameters, such as lag phase duration (or time to threshold, TTT), apparent growth rate (slope), and final fluorescence intensity (plateau or MaxThT) (Figure 2) may correlate with disease burden, severity, or rate of progression [16], opening the door to biomarker‐based patient classification and stratification in clinical trials. Importantly, longitudinal studies are necessary for assessing whether aSyn‐SAA positivity changes over time or in response to disease‐modifying treatments. Some studies have distinguished categorical positivity changes, in which aSyn‐SAA typically remains stable (positive) in established PD or LBD, reflecting persistent pathogenic seeds over time [51, 55, 63]. Another study reported that shortening TTT or increases in slope/MaxThT/AUC signal are associated with faster motor and cognitive decline [51, 55].

In LBD, positive replicates increase longitudinally, while lag phase decreases, predicting dementia onset [55]. In parallel, endpoint‐dilution–based approaches and improved quantitative frameworks now allow estimation of relative seeding activity (e.g., SD50) with sufficient precision to discriminate ~2‐fold differences in aSyn seed burden and to detect multi‐fold reductions after experimental inactivation or over time [64], suggesting that quantitative readouts, not just binary positive/negative classification, may provide biologically and clinically meaningful information for prognosis and therapy monitoring. In research settings, variations in aSyn‐SAAs have proven to be invaluable for studying the biophysical and biochemical properties of aSyn aggregates. By comparing seeding kinetics across different samples or experimental conditions, we can distinguish inter‐disease “strain” differences, such as PD/DLB‐like versus MSA‐like seeding behaviors, which reflect distinct aggregate conformers and tissue tropism. At the same time, aSyn‐SAA readouts capture intra‐disease heterogeneity, in which differences in lag time, growth rate, and fluorescence plateau, along with conformational fingerprints reported in the literature, support the existence of multiple aSyn strain variants within a given clinical syndrome [36, 65, 66]. This has profound implications for our understanding the heterogeneity of synucleinopathies and may help explain differences in clinical course, response to therapy, or neuropathological distribution.

## 3 Diagnostic Performance Across Matrices and Reference Standards

The diagnostic performance of aSyn‐SAA varies depending on the sample type, assay conditions, disease phenotype, and the reference standard against which it is evaluated. In most studies, sensitivity (typically 80%–97%) and specificity (90%–100%) for CSF‐based assays are calculated with respect to a clinical diagnosis of PD or LDB, which is known to be itself imperfect and prone to both under‐ and overdiagnosis, particularly in early or mixed‐pathology cases (Table 1). Peripheral tissue assays, such as those using skin or mucosal biopsies, generally report slightly lower sensitivity but retain high specificity, particularly when standardized processing and sample handling protocols are applied (Table 1). Blood‐based assays have also been reported [67, 68, 69] and are highly desired given the ease in sample accessibility, but are still under active development, as they present challenges related to the complexity of blood samples, the presence of reaction inhibitors, and possible low seed concentration (Table 1).

Importantly, autopsy‐validated cohorts provide a crucial complementary perspective by benchmarking aSyn‐SAA against neuropathology, the gold standard for LBD. In such series, CSF aSyn‐SAA shows near‐perfect sensitivity and specificity for limbic/neocortical Lewy body pathology (e.g., 97%–100% sensitivity and 94%–98% specificity), but reduced sensitivity for earlier or focal stages such as brainstem‐ or amygdala‐predominant disease, where Lewy body burden is lower and pathology may not yet reach cortex or CSF in sufficient quantity [43, 70, 71]. Although these autopsy‐based cohorts are smaller and often include longer intervals between CSF sampling and death, they are indispensable for understanding stage‐dependent performance, clarifying false negatives in prodromal or atypical cases, and refining how aSyn‐SAA results should be interpreted in the context of co‐pathologies (e.g., AD‐LB or amygdala‐predominant Lewy pathology). The variability in assay performance across matrices and disease stages highlights the importance of protocol harmonization and interlaboratory validation, particularly as aSyn‐SAA approaches the ultimate goal of clinical implementation [16]. Moreover, emerging evidence suggests that distinct conformational strains of aSyn may differentially influence seeding kinetics and fluorescence profiles [59, 66, 72, 73], offering opportunities for subtyping synucleinopathies based on biophysical readouts. This concept aligns aSyn‐SAA with the growing field of structural “strainomics”.

### 3.1 Key Analytical Determinants of aSyn‐SAA Performance

The success of aSyn‐SAAs depends critically on the quality, composition, and physicochemical properties of both the substrate and the seed. The interplay between these components dictates the kinetics, efficiency, and specificity of the amplification reaction. Consequently, the choice of recombinant aSyn and the biological source of misfolded seeds are key variables that must be considered when designing or interpreting SAAs.

### 3.2 Recombinant aSyn Substrate: A Major Source of Variability and Reproducibility Risk

Current aSyn‐SAA protocols rely on the use of recombinant human aSyn produced in bacteria as the substrate for amplification. Full‐length wild‐type aSyn [1, 2, 3, 4, 5, 6, 7, 8, 9, 10, 11, 12, 13, 14, 15, 16, 17, 18, 19, 20, 21, 22, 23, 24, 25, 26, 27, 28, 29, 30, 31, 32, 33, 34, 35, 36, 37, 38, 39, 40, 41, 42, 43, 44, 45, 46, 47, 48, 49, 50, 51, 52, 53, 54, 55, 56, 57, 58, 59, 60, 61, 62, 63, 64, 65, 66, 67, 68, 69, 70, 71, 72, 73, 74, 75, 76, 77, 78, 79, 80, 81, 82, 83, 84, 85, 86, 87, 88, 89, 90, 91, 92, 93, 94, 95, 96, 97, 98, 99, 100, 101, 102, 103, 104, 105, 106, 107, 108, 109, 110, 111, 112, 113, 114, 115, 116, 117, 118, 119, 120, 121, 122, 123, 124, 125, 126, 127, 128, 129, 130, 131, 132, 133, 134, 135, 136, 137, 138, 139] is widely used due to its structural fidelity to the native protein. However, certain modifications have been introduced to improve assay sensitivity, reduce background noise, or modulate fibril formation kinetics. For example, the K23Q mutant has been shown to enable more rapid aggregation with some seed types while maintaining specificity [8, 74]. Other variants, such as truncated forms [65], have also been tested in the context of synucleinopathies with distinct conformational profiles. Different laboratories use adaptations of existing protocols for the expression and purification of recombinant aSyn, introducing variability [75, 76, 77]. The presence of even minute amounts of contaminants, such as residual bacterial endotoxins or preformed aggregates in the substrate preparation, can compromise assay reproducibility and increase background noise. Therefore, substrate batches are often subjected to quality control procedures aimed at improving the homogeneity of the sample, such as filtration, ultracentrifugation, or size‐exclusion chromatography, to ensure monodispersity and remove any pre‐aggregated species. In addition, freeze–thaw cycles and prolonged storage at inappropriate temperatures can induce spontaneous oligomerization, emphasizing the importance of handling freshly prepared or aliquoted substrates under standardized conditions. Therefore, including explicit and standardized quality control readouts for the recombinant aSyn substrate, such as assessments of de novo aggregation propensity, batch‐to‐batch variability, and contaminant removal, is essential, yet these parameters are still underreported in many SAA protocol descriptions.

### 3.3 Biological Origin of Seeds: Central Versus Peripheral Readouts

Several types of biological samples have been used as sources of aSyn seeds, each offering specific advantages and limitations. Post‐mortem brain homogenates have been widely used in early‐stage research. They are used as positive controls, allowing for the investigation of regional heterogeneity in aSyn pathology (Table 1 and Table 2). However, their use in clinical diagnostics is limited, as brain tissue is not easily accessible in most neurological disorders. CSF remains the gold standard specimen for diagnostic SAAs due to its direct contact with the brain parenchyma, its relatively low protein complexity, and its established role in biomarker research [8]. In this context, CSF‐derived aSyn seeds have consistently yielded high sensitivity and specificity in differentiating PD and DLB from controls, and from non‐synucleinopathy conditions (Table 1 and Table 2). aSyn‐SAAs using peripheral tissues, such as the olfactory mucosa (OM), skin, and gastrointestinal biopsies, are being increasingly explored as less‐invasive alternatives to CSF (Tables 1 and S1). OM samples, obtained via nasal brushing, are particularly attractive due to their accessibility and due to the occurrence of olfactory alterations (hyposmia) already in pre‐clinical stages of PD [78, 79]. Skin biopsies have also shown promising diagnostic performance. However, lower positivity rates in some cohorts should not be interpreted solely as reduced analytical sensitivity, as they may also reflect genuine biological differences in peripheral involvement. In particular, peripheral seeding activity likely depends on the regional distribution of Lewy pathology, disease stage, and the local aSyn aggregate burden in the sampled tissue, so central‐positive/peripheral‐negative patterns can arise even when the assay itself performs robustly (Table 1). SAAs using blood‐derived samples, including serum, plasma, and extracellular vesicles, offer practical advantages in terms of accessibility and possibility for longitudinal monitoring [69, 80, 81]. However, the lower seed concentration and high protein background pose analytical challenges. Recent studies focusing on blood neuron‐derived extracellular vesicles (nEVs) have shown improved assay performance, suggesting that targeted enrichment strategies may overcome these limitations [82]. Beyond these matrices, tear fluid has emerged as an additional, non‐invasive source of aSyn seeds: total aSyn concentrations are increased in tear fluid of patients with PD compared with controls [83], and tear fluid‐derived aSyn has now been shown to support seeding activity in aSyn‐SAA, with concordant positivity between CSF and tear fluid in a proportion of individuals [84]. These data indicate that tear fluid may provide a practical window onto Lewy body–type pathology that complements CSF, particularly for early diagnosis and longitudinal monitoring.

**TABLE 2:** Sources and volumes of aSyn seeds used in SAAs.

Sample type	Volume (μL)	References
Brain homogenate	2	[24, 27, 28, 32, 64, 85, 86, 87, 88]
Brain homogenate	10	[25]
Brain homogenate	40	[44, 51]
CSF	2	[86]
CSF	5	[28, 41]
CSF	10	[28]
CSF	15	[8, 28, 32, 34, 35, 39, 41, 44, 45, 49, 55, 59, 61, 84, 85, 87, 88, 89, 90, 91]
CSF	40	[20, 37, 46, 47, 48, 50, 51, 54, 56, 57, 63, 92, 93]
Olfactory mucosa	2	[64, 79, 90, 94]
Skin homogenate	2	[64, 86, 88, 95, 96, 97, 98]
Gastrointestinal tract	2	[86, 88, 99, 100]
Saliva	10	[101]
Saliva	5	[102]
Skin homogenate	1	[103]
Blood serum and saliva	Not informed	[67]
Tear fluid	15	[84]

*Note:* Sample types and volumes used as seed sources in aSyn RT‐QuIC assays are listed by category. References include multiple studies using identical conditions.

Abbreviations: μL, microliters; CSF, cerebrospinal fluid.

### 3.4 Pre‐Analytical Variables That Shape Clinical Interpretability

The success of SAA is highly sensitive to preanalytical variables associated with sample collection, processing, and storage, and inconsistent procedures across studies may lead to in *relies on spontaneous misfolding* inter‐laboratory variability and influence assay performance. CSF should be collected following standard lumbar puncture protocols, with particular attention to minimizing or excluding visible blood contamination, as even low levels of hemoglobin can inhibit aggregation and distort fluorescence readouts. To this point, many groups either discard heavily blood‐stained samples or apply early hemoglobin cutoffs to define acceptable specimens. After collection, CSF should be promptly processed, centrifuged to remove cells and debris, aliquoted, and stored at −80°C in low‐protein‐binding tubes, avoiding repeated freeze–thaw cycles. Multiple large cohort studies have shown that banked, long‐term‐frozen CSF remains a robust matrix for aSyn‐SAA when these conditions are met, enabling retrospective analyses and multicenter biomarker efforts [16, 20, 104, 105].

For peripheral tissues, such as skin and mucosa biopsies, standardized dissection and homogenization protocols, including defined biopsy depth/site, controlled tissue‐to‐buffer ratios, and bead‐beating or sonication steps, are essential to reproducibly release embedded aSyn aggregates without introducing excessive mechanical or proteolytic damage [94, 95]. Blood‐derived matrices (serum, plasma, extracellular vesicles) pose additional challenges because they contain high concentrations of proteins (including lipoproteins) and lipids that can bind aSyn, interfere with ThT fluorescence, or act as aggregation inhibitors [106]. Similar inhibitory effects have been demonstrated for CSF lipoproteins [46], which suppress seeding, underscoring the need for dilution schemes or enrichment strategies that mitigate these matrix effects.

Seed input volume and dilution must also be carefully optimized. Excess seed can saturate the reaction, compressing lag times and plateauing fluorescence, whereas underloading increases the risk of false negatives [8]. The choice of dilution buffer, its compatibility with the assay mix, and the presence of potential inhibitors (e.g., hemoglobin, lipoproteins, proteases, or lipids) all require explicit control and reporting to support reproducible SAA performance across laboratories.

### 3.5 Reaction Environment as a Determinant of Seeding Kinetics and Specificity

The composition of the reaction buffer in aSyn‐SAAs plays a pivotal role in modulating the seeding kinetics, protein stability, and fluorescence readout of the assay. Unlike conventional aggregation assays, aSyn‐SAA is designed to amplify seed‐dependent templated misfolding. Therefore, assay conditions must minimize spontaneous substrate aggregation to avoid false‐positive signals while enabling efficient seed‐driven conversion and fibril amplification. Subtle differences in buffer components, such as salt concentration, pH, and the presence of small molecules can drastically alter the nucleation efficiency, aggregation pathways, and assay specificity.

Phosphate‐based buffers are the most used systems across published protocols, typically with concentrations ranging from 40 to 140 mM and pH values between 7.5 and 8.2 (Table 3). This buffering system offers high compatibility with recombinant protein preparations, maintains a stable pH over the assay duration, and supports the electrostatic environment conducive to amyloid formation. However, phosphate has poor buffering capacity above pH 7.5 and can precipitate polyvalent cations [107].

**TABLE 3:** Buffer and salt conditions reported used in aSyn‐SAA.

Buffer	[Buffer] (mM)	pH	[NaCl] (mM)	References
PBS	10	7.4	500	[73]
PBS	40	8.0	500	[27, 34, 79, 86, 89, 96]
PB	40	8.0	170	[8, 28, 35, 41, 49, 55, 59, 61, 64, 84, 85, 88, 91, 95, 97, 98, 100]
PB	40	8.0	*	[25, 26]
PB	40	8.0	170/500	[44]
PB	40	8.2	160	[99]
PB	100	8.2	170	[32]
PB	100	8.2	—	[24, 28, 32, 39, 41, 90, 94]
PB	100	8.2	100	[67]
PB	100	8.5	—	[101]
PB	100	7.5–8.0	0–170	[69]
PB	100	7.5–8.0	50	[113]
PB	140	8.0	600	[87]
Tris	10	7.5	—	[103]
Tris	10	7.6	—	[114]
Tris	50	7.4	250	[115]
PIPES	100	6.5	500	[20, 36, 37, 46, 48, 51, 56, 57, 63, 93]
PIPES	100	6.5	440	[54]
PIPES	100	7.0	—	[45]
PIPES	100	6.5	455	[47]

*Note:* Studies were grouped by buffer and ordered by publication year. In cases with more than one salt concentration per study, all are listed.

Abbreviations: mM, millimolar; PB, phosphate buffer; PBS, phosphate‐buffered saline.

* Na_3_Citrate.

In some cases, Good's buffers, such as PIPES, have been employed (Table 3). These buffers were specifically designed to provide high buffering capacity and minimal interaction with biomolecules at physiological pH, thereby offering more stable control of proton activity over extended incubation periods and temperature cycles [107, 108]. The choice of buffer may affect the surface charge and solubility of aSyn monomers, impacting their propensity to aggregate under seeding conditions. Tris‐based buffers are employed, though less frequently (Table 3). The choice of buffer may affect the surface charge and solubility of the aSyn monomers, impacting their propensity to aggregate under seeding conditions. Furthermore, pH influences the protonation state of amino acid residues, which can modulate intermolecular interactions during fibril elongation. Lower pH values may slow down aggregation, whereas slightly alkaline conditions tend to favor faster nucleation [109, 110], although this may occur at the cost of reduced specificity.

Salt concentration, especially NaCl, is another variable of interest. High ionic strength can shield repulsive charges between protein monomers, facilitating closer interactions and promoting aggregation [111]. Most protocols include NaCl in the range of 100–600 mM (Table 3), although some protocols explore the use of kosmotropic or chaotropic salts to fine‐tune the aggregation dynamics [112].

### 3.6 Fluorescent Probes: ThT and Beyond

The real‐time monitoring of fibril formation in SAAs relies on the fluorescence enhancement of amyloid‐binding dyes, most commonly ThT. Upon binding to cross‐β‐sheet structures, ThT exhibits a characteristic shift in its excitation and emission profiles (typically 450/480 nm), enabling real‐time quantification of fibril growth [116].

The concentration of ThT must be carefully optimized, typically ranging from 5 to 20 μM, since excessively high levels can quench the signal or interfere with aggregation kinetics [116, 117, 118]. Some studies suggest that ThT may preferentially bind to certain fibril polymorphs [119, 120], which could influence not only signal intensity but also the detection bias toward specific aSyn strains.

Alternative probes, such as luminescent conjugated oligothiophenes (LCOs), have been explored for their capacity to distinguish between different amyloid conformers based on spectral fingerprinting [121]. Although not yet standardized in SAA protocols, LCOs may offer opportunities for multiplexed detection or subtype discrimination.

### 3.7 Additives and Modulators

Several protocols incorporate additional reagents intended to modulate aggregation or improve assay robustness. These may include detergents (e.g., SDS, Triton X‐100), reducing agents (e.g., DTT, TCEP), and chelators (e.g., EDTA) [104, 113]. It is also essential to consider the type and number of beads used for mechanical agitation, as their interaction with the buffer can influence shearing forces, temperature distribution, and localized aggregation [122, 123, 124].

### 3.8 Impact of Buffer Systems on Aggregation Kinetics and Assay Sensitivity

Altogether, the buffer system defines the biophysical landscape in which the aggregation reaction occurs. Inadequate buffer conditions may delay the lag phase, yield low‐intensity ThT signals, or promote off‐pathway aggregates that lack diagnostic relevance. Conversely, optimal buffers accelerate the conversion of monomers into amyloid fibrils with distinct, reproducible kinetics that reflect the seeding capacity of the sample.

Comparative analyses have shown that protocols using similar aSyn substrates and sample types can yield different diagnostic outcomes depending on buffer formulation [104, 113]. This underscores the importance of not only selecting a buffer based on precedent but also validating and, if needed, re‐optimizing buffer components for specific experimental contexts, such as when switching between sample types (e.g., CSF vs. mucosa) or adapting protocols across laboratories.

### 3.9 Standardization Challenges and Future Directions

Despite the central role of buffers and reagents in assay fidelity, there is currently no universally accepted buffer formulation for aSyn‐SAA. Ongoing efforts toward protocol harmonization should include side‐by‐side comparisons of buffer systems under blinded, multicenter conditions. The inclusion of well‐defined internal controls, reference standards, and lyophilized buffer kits may further enhance assay reproducibility and facilitate its clinical translation.

### 3.10 Physical Parameters Governing Amplification Dynamics

In aSyn‐SAAs, the physical environment of the reaction is as critical as the biochemical components. The dynamic process of amyloid formation is enhanced not only by the intrinsic properties of the substrate and seed but also by mechanical agitation, temperature, and reaction duration. These parameters collectively define the energy landscape that governs nucleation, fibril elongation, and fragmentation, three essential phases of the seed amplification cycle.

### 3.11 Beads: Type, Size, and Quantity

One of the defining features of aSyn‐SAA is the use of beads within reaction wells to promote mechanical agitation. Beads facilitate the fragmentation of growing fibrils into smaller seeds, thereby enhancing the exponential amplification process [122, 123, 124]. Different protocols employ beads of varying materials, sizes, and quantities, each of which influences the efficiency and reproducibility of the assay.

The most commonly used beads are: (i) Zirconium/silica beads (0.1–0.5 mm) (Table 4)—these are dense and inert, and provide strong shearing forces and are widely available; (ii) Silica or glass beads (0.8–3 mm) (Table 4): these are preferred in some protocols for their compatibility with specific agitation systems or well formats.

**TABLE 4:** Mechanical and incubation conditions used in aSyn‐SAAs.

Bead material	Diameter (mm)	Quantity	Agitation (rpm)	Incubation (min)	Temperature (°C)	References
Silica	0.8	6 beads	400	1	42	[8, 28, 35, 49, 55, 59, 61, 64, 84, 85, 86, 88, 91, 98, 100]
Silica	0.8	6 beads	400	1	33/42	[41]
Silica	0.8	6 beads	400	1	30/42	[97]
Silica	2	1 bead	—	—	37	[113]
Glass	3	—	600	14	42	[79]
Glass	3	—	600	29	37	[102]
Glass	0.8	6 beads	200	14	30	[89]
Glass	0.	6 beads	400	1	42	[27, 34]
Glass	1.0–1.2	4 beads	400	1	37	[24, 99]
Glass	0.5	3 mg	200	14	30	[94]
Glass	0.5	3 mg	200	14	30/42	[90]
Zirconium/silica	0.1	3 mg	400	1	33/42	[41]
Zirconium/silica	0.5	3 mg	200	14	30	[28, 32]
Zirconium/silica	0.5	3 mg	200	14	30/42	[39]
Zirconium/silica	0.5	3 mg	200	14	30	[69]
Silica nitride	—	1 bead	800	29	37	[20, 46, 47, 48, 50, 51, 63]
Silica nitride	—	2 beads	800	29	42	[56, 57]
Silica nitride	—	2 beads	800	14	42	[54]
Silica nitride	3,2	2 beads	800	15	42	[93]
No beads	—	—	500	29	37	[37, 92]
No beads	—	—	400	1	42	[95]
No beads	—	—	200	14	30/42	[90]
No beads	—	—	500	15	31.2	[101]
No beads	—	—	500	1	40	[45]
No beads	—	—	400	14	37	[25]
No beads	—	—	2000	12	37	[115]
No beads	—	—	500	15/1	37/42	[44]
No beads	—	—	400	14	42	[26]
No beads	—	—			37	[58]
No beads	—	—			50	[103]
No beads	—	—	500	14	24	[126]
No beads	—	—			40	[67]

The number of beads used per well also varies. Some protocols specify exact numbers (e.g., six 0.8 mm beads), while others use fixed masses (e.g., 3 mg per well) [109] (Table 4). Excessive mechanical agitation (e.g., overly aggressive shaking or bead‐beating) can promote prion‐independent conversion of the recombinant substrate and premature ThT signal increase, whereas insufficient mechanical energy reduces fibril fragmentation and leads to longer lag phases in SAA‐type assays [110, 125].

### 3.12 Agitation Method and Equipment

Mechanical agitation is crucial for inducing fibril fragmentation and promoting continuous seed generation. Typically, reactions are performed in 96‐well or 384‐well optical plates, which are placed in microplate readers or orbital shakers equipped with precise temperature and shaking controls.

The most common systems include multimode plate readers with programmable shaking and real‐time fluorescence reading, as well as orbital shakers or double‐orbital mixers [104, 109, 110], which are used in protocols that perform fluorescence measurements offline.

Agitation is typically performed in cyclical patterns, consisting of 1 min of shaking at 200–800 rpm, followed by 1–29 min of rest or incubation (Table 4). These cycles are repeated continuously for 48 to 120 h, depending on the assay protocol. The intensity and frequency of shaking have a direct impact on fibril breakage and aggregation kinetics [110, 125].

Protocols must be calibrated so that mechanical shear is sufficient to support exponential amplification but not so intense that it damages monomeric substrate or disrupts signal detection. Additionally, differences in shaker calibration across laboratories may contribute to variability in assay results, underscoring the need for equipment standardization.

### 3.13 Temperature and Reaction Time

Temperature is another crucial parameter, as it affects protein folding, fibril stability, and dye fluorescence [8, 109, 127]. Most SAAs for aSyn are performed at 30°C to 42°C, with the majority favoring the upper end of this range (Table 4). A temperature of 42°C is commonly chosen to accelerate aggregation kinetics while preserving substrate integrity and avoiding heat‐induced misfolding artifacts.

Reaction duration varies substantially across protocols and has evolved over time. Earlier assays often required 60–120 h [8, 28, 32], whereas newer optimized aSyn‐SAA formats can yield results within 24 h [54, 56, 57, 88], depending on sample type, protocol design, and readout criteria. Shorter run times may improve feasibility for clinical implementation and larger‐scale studies, although assay duration can still be extended to enhance sensitivity for low‐seeding samples.

A crucial consideration is the temporal resolution of ThT readings, which are typically taken every 15–45 min during the assay. This enables detailed kinetic curve analysis, providing information on the lag phase, exponential growth rate, and fluorescence plateau, all of which contribute to characterizing the seeding profile (Figure 2).

## 4 Cohort Composition and Diagnostic Performance

Although technical parameters, such as buffer composition, seed volume, and agitation protocol, are critical for aSyn‐SAA performance, the demographic characteristics of the study cohorts also play a fundamental role in determining assay sensitivity, specificity, and clinical applicability. An integrated evaluation of published protocols reveals that differences in age, sex distribution, disease subtype, and disease progression stage may impact the outcome of aSyn‐SAA assays.

### 4.1 Sample Size and Diagnostic Group Stratification

Most studies evaluated here include between 20 and 200 participants, with varying proportions of patients and controls (Table S1). Well‐balanced case–control designs tend to yield more reliable estimates of sensitivity and specificity. Conversely, smaller or unbalanced cohorts may inflate diagnostic accuracy due to spectrum bias and limit the stability of subgroup analyses. At the same time, these small‐to‐moderate studies have been crucial for method development, protocol optimization, and initial exploration of assay performance in selected clinical phenotypes.

More recently, large multicenter cohorts have substantially expanded the evidence base for clinical translation. In particular, the Parkinson's Progression Markers Initiative (PPMI) analysis of 1123 individuals (545 PD, 163 healthy controls, 54 SWEDD, 51 prodromal RBD/hyposmia, and 310 non‐manifesting LRRK2/GBA carriers) demonstrated high diagnostic accuracy for sporadic PD and enabled robust assessment of genetic, clinical, and prodromal subgroups in a deeply phenotyped, international cohort [48]. These large‐scale data complement earlier, smaller studies by confirming assay sensitivity and specificity in real‐world, heterogeneous populations and by providing adequately powered stratified analyses, thereby strengthening the rationale for incorporating aSyn‐SAA into biomarker frameworks and trial designs.

### 4.2 Age and Sex Distribution

Several studies report participant age ranges or mean ages, with a concentration in individuals over 60 years (Table S1). This is expected given the epidemiology of synucleinopathies. However, age‐related differences in aSyn metabolism, proteostasis, and neurodegeneration may influence seeding activity in biological samples. For example, elderly individuals, even healthy controls, may harbor low levels of misfolded aSyn, which could affect assay specificity.

Sex‐based analysis is rarely addressed in detail, although some studies report distributions (Table S1). Biological differences in immune response and hormonal regulation may contribute to subtle variations in seeding dynamics. Further investigation is warranted to determine whether sex influences aSyn‐SAA outcomes.

### 4.3 Diagnostic Subtypes and Pathological Heterogeneity

Diagnostic diversity across cohorts, such as inclusion of PD, DLB, and MSA, may influence aSyn‐SAA reactivity and must be considered when interpreting assay performance. Notably, while PD and DLB samples generally show high positivity rates, the sensitivity of aSyn‐SAA for MSA is lower in most protocols, ranging from 6% to 85% [35, 128, 129]. This pattern likely reflects differences in aSyn strain conformation, cellular host (neuronal versus oligodendroglial), and regional pathology distribution between Lewy body disorders and MSA.

Importantly, several groups have now shown that protocol choice can flip this picture for MSA. Assays specifically tuned to MSA‐type strains (e.g., using modified buffer, substrate, temperature, and readout criteria) can detect MSA CSF with high sensitivity and distinguish “MSA‐type” kinetic profiles from “LBD‐type” curves in the same platform [61, 93].

Taken together, these findings strengthen the idea that aSyn‐SAAs are not generic “yes/no” tests for synucleinopathy, but conformation‐ and context‐sensitive assays whose diagnostic window is defined by their biochemical parameters. The consistent ability of some protocols to both detect MSA aggregates and distinguish their kinetic signatures from those of PD/DLB, contrasted with the near‐complete insensitivity of other, PD‐optimized assays to MSA, directly supports the notion that protocol design is crucial for outcomes and reliable subtyping of synucleinopathies in clinical and translational settings.

## 5 Reproducibility Across Laboratories: What Has Worked So Far

Despite the growing number of studies demonstrating the utility of aSyn‐SAAs for detecting aSyn seeding activity, one of the most significant barriers to broader adoption, especially in clinical settings, is the lack of standardization across laboratories.

Variability in mechanical and incubation conditions is a primary source of inconsistency across SAA studies. Even minor differences in shaker type, well format, plate sealing, and instrumentation can affect airflow, evaporation, and heat transfer, all of which influence fibril formation.

Therefore, detailed reporting of these parameters is essential for reproducibility and scalability, which will be required for broad regulatory approval.

## 6 Inter‐Laboratory Quality Control and Benchmarking

To address the need for harmonization, several strategies can be proposed:

Reference standards: The development and distribution of lyophilized control seeds (e.g., synthetic fibrils or brain homogenates with defined activity) would enable benchmarking across centers and facilitate calibration of kinetic parameters.

- Ring trials (proficiency testing): Comparative “round‐robin” studies, in which the same blinded CSF panel is tested independently in multiple laboratories using distinct SAA protocols and recombinant substrates, have already shown high qualitative agreement and systematic, rather than random, differences in kinetic readouts, highlighting the feasibility and value of formal ring trials for aSyn‐SAA [32, 41, 130, 131].
- Clinical panels and shared resources: Rather than proposing centralized distribution of primary patient material, which may be constrained by ethical and practical limitations, we suggest leveraging existing well‐established cohorts and biorepositories (e.g., PPMI, BioFIND, BioFINDER) to enable replication, cross‐platform comparisons, and blinded multicenter validation. In addition, inter‐laboratory proficiency panels may incorporate standardized synthetic materials and carefully curated clinical samples under appropriate governance frameworks.
- Harmonized SOPs: Detailed and freely available standard operating procedures (SOP), including specifications for sample handling, reagent preparation, equipment calibration, and data interpretation, are vital for consistent assay implementation. Recently published consensus‐type methods protocol articles provide step‐by‐step Syn‐SAA workflows for CSF [20], including guidance on quality control of recombinant substrate, plate layout, positivity thresholds, and kinetic analysis, which can serve as templates for harmonized SOPs and accreditation schemes.

## 7 Regulatory and Translational Perspectives

The ultimate goal of standardization is to support the regulatory approval of aSyn SAA as a diagnostic tool, whether for early detection of PD, differential diagnosis of synucleinopathies, or monitoring therapeutic efficacy. Collaborations among academic consortia, biotech developers, clinical neurologists, and regulatory bodies will be crucial in bridging the gap between experimental success and clinical implementation. Lessons learned from the journey of prion RT‐QuIC toward clinical use can serve as a roadmap, highlighting the importance of multicenter validation, cross‐border regulatory harmonization, and sustained investment in infrastructure and training.

## 8 Key Considerations for Clinical and Translational Implementation

As aSyn‐SAA continues to gain traction in both research and diagnostic settings, many laboratories are now considering adopting or adapting the technique. However, due to the assay's sensitivity to multiple experimental variables, careful planning and execution are essential to ensure success.

## 9 Critical Checkpoints Before Starting

Before initiating the use of aSyn‐SAAs, new users should evaluate and secure the following components:

- *Infrastructure and Equipment*
  ○ Access to a fluorescence microplate reader with precise temperature control (±0.5°C), capable of shaking and continuous readout over 60–120 h.
  ○ An orbital shaker (if using offline readout), compatible with microplates and able to sustain set rpm ranges (200–600 rpm).
  ○ Controlled‐temperature incubators and low‐binding consumables (e.g., protein LowBind tubes, low‐evaporation plates).
- *Protein Production Capability or Supply*

Recombinant human aSyn must be available in a sufficiently pure form. New users without in‐house protein purification capabilities may consider collaborating with protein core facilities or sourcing from specialized vendors. Given that recombinant aSyn is both the core substrate and a major source of variability [75, 76, 77], each new protein batch should undergo rigorous QC before any clinical or translational use. Routine checks should include purity and monomeric state (e.g., by SDS–PAGE and size‐exclusion or DLS where available), absence of pre‐formed aggregates after thawing, and reproducible seeding responsiveness using a small panel of well‐characterized positive and negative controls. To mitigate the risk of spontaneous aggregation, laboratories should implement pilot plates containing only buffer, a negative‐control sample, and substrate (no added seeds) to establish baseline noise and confirm that no wells exceed the fluorescence threshold throughout the assay duration. Any batch that shows frequent spontaneous positives or erratic baseline drift should be discarded. Before testing clinical samples, it is advisable to conduct several full validation experiments with that substrate lot to confirm consistent lag times and maximum fluorescence for positive controls, and a stable low signal for all negatives.

- *Sample Handling and Biosafety*
  ○ Establish SOPs for handling human biological materials (e.g., CSF, skin biopsies, nasal swabs), including obtaining ethical approvals and implementing biosafety protocols.
  ○ Ensure availability of Class II biosafety cabinets and secure −80°C storage for sample preservation.
- *Controls and Validation Material*
  ○ Include positive controls (e.g., aSyn fibrils or known positive CSF) and negative controls (healthy donor CSF or buffer only) in each run.
  ○ Perform initial runs with test samples in triplicate or quadruplicate to assess assay variability.

## 10 Interpretation of Results

New users should adopt a structured approach to result analysis:

- Define threshold fluorescence empirically using multiple negative controls (e.g., mean + 5 SD of background fluorescence).
- Monitor kinetic curves for the duration of the lag phase, the exponential slope, and the plateau height.
- Consider a sample positive if ≥ 2 of 3–4 technical replicates cross the fluorescence threshold within the set time frame.
- Include blinded replicate runs to evaluate intra‐assay and inter‐assay consistency.
- Avoid relying solely on endpoint fluorescence; the kinetic profile can offer insights into seed strength, strain differences, and technical quality.

## 11 Conclusions

The adaptation of SAA to detect aSyn seeding activity has profoundly transformed the diagnostic landscape of synucleinopathies. By enabling the ultrasensitive detection of misfolded aSyn aggregates in accessible biological samples, aSyn‐SAA bridges the gap between molecular pathology and clinical neurology, opening up the possibility for disease classification [18]. The assay's high specificity and sensitivity position it as one of the most promising tools for in vivo diagnosis, early disease detection, and subtype discrimination among PD, DLB, and other aSyn‐related disorders.

Beyond its diagnostic potential, aSyn‐SAAs offer a unique window into the biophysical and strain‐specific properties of aSyn aggregates. Kinetic curve analysis, combined with structural investigations, suggests that distinct conformers or strains of aSyn may underlie the clinical heterogeneity observed in synucleinopathies. This opens the door also to novel molecular classification systems based not only on pathology distribution but also on the conformational and seeding characteristics of the aggregates, a concept known as strainomics. Strikingly, the ability of aSyn‐SAAs to distinguish between aSyn strains with subtle biophysical differences is likely to impact the development of personalized therapies.

As aSyn‐SAAs protocols become more refined and adapted to different biological matrices, including OM, skin, and blood‐derived samples, their utility in minimally invasive diagnostics increases. The ability to detect seeds in preclinical or prodromal stages could transform current diagnostic workflows, enabling earlier intervention and more accurate patient stratification for clinical trials. Moreover, the quantitative and kinetic nature of the assay offers the potential for monitoring disease progression or therapeutic response, although these applications remain to be validated in longitudinal studies.

Despite these advances, challenges remain in achieving widespread clinical implementation. These include protocol standardization, inter‐laboratory reproducibility, and regulatory validation. Nonetheless, the foundational evidence generated in recent years provides strong momentum toward integration into clinical neurology.

In conclusion, aSyn‐SAA represents a gateway to understanding the molecular diversity, progression, and therapeutic vulnerability of synucleinopathies. As the field continues to evolve, interdisciplinary collaboration between biochemists, biophysicists, neurologists, pathologists, and bioengineers will be essential to fully exploit the impact of aSyn‐SAA in both research and clinical care. With continued refinement, standardization, and integration into diagnostic pipelines, aSyn‐SAAs stand as a cornerstone of the next generation of precision diagnostics in neurodegenerative diseases that extend beyond the typical synucleinopathies, as aSyn copathology is also common in other diseases such as AD.

## Author Contributions

Manuela Amaral‐do‐Nascimento and Daniela F. Santos performed literature searches and wrote the manuscript. Tuane C.R.G. Vieira and Tiago F. Outeiro conceived and wrote the manuscript. All authors read and approved the final manuscript.

## Funding

This work was supported by the Coordenação de Aperfeiçoamento de Pessoal de Nível Superior—Brasil (CAPES)—Finance Code 001, grants from the National Council for Scientific and Technological Development (Grants 408046/2021‐0 and 409103/2023‐3), and the Fundação Carlos Chagas Filho de Amparo à Pesquisa do Estado do Rio de Janeiro for research support in the state of Rio de Janeiro (FAPERJ) (Grant E‐26/204.253/2024). Fundação para a Ciência e Tecnologia (FCT) 2023.15347.PEX.

## Conflicts of Interest

The authors declare no conflicts of interest.

## Supporting information


**Table S1:** Diagnostic groups and demographic characteristics of aSyn SAAs.

## Data Availability

This manuscript is a systematic review and synthesis of previously published studies. No new primary datasets were generated. All data supporting the findings of this review are available within the article and its Supporting Information and/or the cited literature.
