# Supplementary material for: A Systematic Comparison of Alpha‐Synuclein Seed Amplification Assays for Increasing Reproducibility
Source: Ann Clin Transl Neurol. 2026 Apr 1;13(6):1088–105. doi: 10.1002/acn3.70384 (PMC13251439; doi:10.1002/acn3.70384)
Supplement: Supplementary file 1 — Table S1: Diagnostic groups and demographic characteristics of aSyn SAAs. [file ACN3-13-1088-s001.docx]

**Supplementary Table 1. Diagnostic groups and demographic characteristics of aSyn SAAs.**

| Diagnostic Group | | Demographics | | aSyn SAA | Reference |
| --- | --- | --- | --- | --- | --- |
| Stratification | **Sample size** | **Age**  **(mean age, years)** | **Gender**  **(male %)** | **aSyn seeds source** |  |
| PD, DLB, CBD, HC | 30  (10 DLB, 10 PD, 10 HC) | DLB: 77.9  PD: 78.1 | DLB: 50  PD: 70  HC: 70 | Brain | ^30^ |
| PD, DLB, AD, HC | 45  SNc from 11 PD, 11 HC  basal ganglia from 5 DLB, 10 AD, 8 HC | PD: 82.5 ± 6.7  HC: 81 ± 7.6  DLB: 83.6 ± 7.7  AD: 83.6 ± 6  HC: 86.25 ± 8.7 | PD: 90.9  HC: 72.7  DLB: 60  AD: 40  HC: 50 | Brain | ^31^ |
| DLB, HC | 13  (7 DLB, 6 HC) | DLB: 65.7  HC: 63.3 | DLB: 85.7  HC: 66.7 | Brain | ^32^ |
| MSA, PD, other aSynopathies | 40    (15 MSA, 15 PD, 5 supranuclear progressive palsy, 5 controls) | 72.3 | 47.5 | Brain | ^33^ |
| MSA, LBD, HC | 9  (2 MSA, 6 LBD, 1 HC) | 69.5 | 33.3 | Brain | ^34^ |
| human prion diseases (including sporadic and genetic CJD, GSS)  AD, DLB | 6 naturally CWD-infected, 6 naturally infected captive Rocky Mountain elk | not specified | not specified | Brain homogenates | ^35^ |
| OPTIMA and Oxford Discovery cohorts    DLB, PD, PSP, AD | 99 from OPTIMA cohort   (12 DLB, 2 PD, 2 PSP, 3 corticobasal degeneration, 17 DLB with AD, 13 AD with incidental LBs, 30 AD, 20 HC)  38 from Oxford Discovery cohort   (20 PD, 15 controls, and 3 at‐risk) | OPTIMA cohort: 74.2  Oxford cohort:  PD: 67.9 ± 9.3  HC: 64.3 ± 9.1 | OPTIMA cohort: 39         PD: 63  HC: 36.4 | CSF | ^89^ |
| Kassel cohort   PD, DLB, MSA ND, AD, other neurological disorders | 193  (76 PD, 10 DLB, 10 MSA + 65 with other neurologic diseases, 14 AD, 18 ND) | 66.8 | 26 | CSF | ^37^ |
| PD, DLB, AD, HC | 60   (12 PD, 17 DLB, 16 AD, 15 HC) | PD, DLB, AD: 71.8    HC: 70.5 | 73.3 | CSF | ^8^ |
| Leucine-rich kinase 2 (LRRK2)-linked Parkinson's disease (PD) | 51  (15 LRRK2 p.G2019S PD, 10 IPD, 16 LRRK2 p.G2019S NMC, 10 HC) | Not specified | Not specified | CSF | ^38^ |
| PD, HC | 61   (44 PD, 17 HC) | 66 | Not specified | CSF | ^39^ |
| BioFIND study  Moderate‐to‐advanced PD, HC | 184  (105 PD, 79 HC) | PD: 68   HC: 65.6 | PD: 62.7   HC: 51.1 | CSF | ^40^ |
| PD, HC | 170   (118 PD, 52 HC) | PD: 61 ± 1   HC: 64 ± 9 | PD: 64  HC: 58 | CSF | ^41^ |
| BioFIND study    Moderate‐to‐advanced PD, HC | 193    (108 PD, 85 HC) | PD: 68   HC: 65.6 | PD: 62.7   HC: 51.1 | CSF | ^42^ |
| PD, DLB, MSA, iRBD, PAF | 528   (21 LB-α-Syn+, 48 DLB, 17 Dementia with incidental LB, 101 LB-α-Syn−, 17 AD, 31 PSP or PSP/CBD, 33 MSA, 71 PD, 28 PAF, 18 iRBD, 143 Controls) | 65.3 | 58.5 | CSF | ^43^ |
| PD, MSA, HC | 225  (94 PD, 75 MSA, 56 HC) | PD: 66.78  MSA: 59.67  HC: 61.14 | PD: 62.7  MAS: 74.6  HC: 50 | CSF | ^27^ |
| Mayo Longitudinal ASynopathy Biomarker Study        MSA, PD, DLB, HC | Discovery: 24 MSA, 14 HC; Confirmatory: 38 MSA, 16 PD, 13 DLB, 15 HC | MSA: 59.2 ± 6.8  PD: 65.9 ± 6.0  DLB: 66.8 ± 8.3  HC: 58.5 ± 7.4 | MSA: 43    PD: 15    DLB: 13   HC: 14 | CSF | ^44^ |
| PD, DLB, asymptomatic mutation carriers, HC | 236 PD, 49 DLB, 14 asymptomatic mutation carriers, 26 HC | PD: 64 ± 9  DLB: 72 ± 7  Asymptomatic mutation carriers: 58 ± 15  HC: 59 ± 12 | PD: 65  DLB: 70  Asymptomatic mutation carriers: 36  HC: 54 | CSF | ^45^ |
| IRBD, HC | 103   (52 IRBD, 40 HC, 11 neurological controls (NC)) | IRBD: 71.3  HC: 69.7 NC: 61.3 | IRBD: 82.7  HC: 70  NC: 81.8 | CSF | ^46^ |
| ISNB and VUmc cohorts    MCI, HC | 289   (231 mild cognitive impairment MCI, 58 controls) | MCI: 68.6  HC: 67.3 | MCI: 70.6  HC: 79.5 | CSF | ^47^ |
| Parkinson’s Progression Markers Initiative (PPMI) study protocol (n=683)    PD, HC, SWEDD | 80   (30 PD, 30 HC, 20 SWEDD) | PD: 62.1 ± 9.3  HC: 63.8 ± 10.6  SWEDD: 59.6 ± 10.6 | PD: 63.3  HC: 60  SWEDD: 60 | CSF | ^48,133^ |
| PD, MSA, PSP, CBD, HC | 112   (20 PD, 37 MSA, 23 PSP, 13 CBD, 19 HC) | CS: 72.2  PD: 70.5   MSA: 62.5  PSP: 74.5   CBD: 70.3 | CS: 42.1   PD: 45  MSA: 54.1   PSP: 60.9   CBD: 23.1 | CSF | ^49^ |
| BioFINDER Study    AZSAND/BBDP cohort  PD, PDD, MSA, PSP, Controls | 141   (50 PD, 14 PDD, 15 MSA, 15 PSP, 47 Controls) | PD: 68.0  PDD: 71.1  MSA: 66.6  PSP: 72.3  HC: 66.6 | PD: 50  PDD: 57  MSA: 60  PSP: 47  HC: 43 | CSF | ^50^ |
| PD, HC | 193   (111 PD, 82 HC) | PD: 60.8   HC: 57 | PD: 59.5   HC: 40 | CSF | ^51^ |
| OPTIMA and Oxford Discovery cohorts       PD, MSA, iRBD, HC | 198    (74 PD, 24 MSA, 45 iRBD, 55 HC) | PD: 65.3 ± 9.0  IRBD: 65.7 ± 8.4  MSA: 63.8 ± 8.2  HC: 76.4 ± 11.9 | PD: 64.9   iRBD: 73.3   MSA: 58.3   HC: 50.9 | CSF | ^52^ |
| PD, HC | 14   (4 PD, 10HC) | PD: 71   HC: 52.5 | 77.4 | CSF | ^53^ |
| PD, MSA, CBD, PSP, HC | 129   (55 PD, 27 MSA, 7 CBD, 16 PSP, 24 HC) | 69.1 | 55.8 | CSF | ^54^ |
| PD, HC | 1123  (545 PD, 163 HC, 54 SWEDD, 51 prodromal, 310 NMC) | PD: 63.4  HC: 62.6  SWEDD: 63.3  Prodomal: 67.7  NMC: 61.7 | PD:62  HC: 66  SWEDD: 59  Prodomal: 78  NMC: 43 | CSF | ^55^ |
| PD | 231 PD | Not specified for overall cohort | Not specified for overall cohort | CSF | ^56^ |
| DeNoPa cohort  PD, iRBD, HC | 233    (113 de novo PD, 64 HC, 29 iRBD) | PD: 63  iRBD: 66.9  HC: 66 | PD: 73.2  iRBD: 72.7  HC: 70 | CSF | ^57^ |
| Probable and possible DLB, HC | 290   (191 DLB, 50 matched controls, 49 analytical controls) | 69.9 ± 6.8 | 85 | CSF | ^25^ |
| LBD, LBD-, HC | 37  (8 LBD+, 23 LBD-, 6 HC) | LBD+: 59.7  LBD-: 56.3  HC: 51 | LBD+: 75  LBD-: 26  HC: 66.7 | CSF | ^58^ |
| Munich and Tübingen DIAN study sites  AD | 29 mutation non‐carriers, 26 asymptomatic mutation carriers, 15 symptomatic mutation carriers | Asymptomatic: 35.2  Mutation non-carriers: 34.6 | Not specified | CSF | ^59^ |
| AD | 417 AD | 65.4 | 35 | CSF | ^60^ |
| BioFINDER-1  LBD, PD/CU, PD/PDD, CI/LBD, CI/AD | 196 | 69.8 | 66.8 | CSF | ^24^ |
| ADNI database  CU, MCI, AD | 1637 | 77 | 58.3 | CSF | ^61^ |
| PSP, CBD, IDT | 106   (59 PSP, 37 CBD, 10 IDT) | PSP: 69   CBD: 68.2   IDT: 72.1 | PSP: 58   CBD: 30   IDT: 30 | CSF | ^62^ |
| Systemic ASyn Sampling Study (S4) cohort  PD, HC | 71  (50 PD, 21 HC) | 63.0 ± 8.0 | 60.6 | CSF | ^63^ |
| PD, HC | 54    (34 PD, 20 controls) | 60.4 | 60 | CSF | ^64^ |
| UK parkinsonism cohort  PPMI cohort  Tübingen cohort  PD, PSP, HC | 1631  (1331 PD, 52 PSP, 248 HC) | Not specified | Not specified | CSF | ^65^ |
| MSA, PD, PSP, HC | 249 (114 MSA, 49 PD, 40 PSP, 46 HC) | MSA: 62.4 ± 8.1 PD: 62.1 ± 8.9 PSP: 73.0 ± 7.7 HC: 21.5 ± 12.8 | Not specified | CSF | ^66^ |
| PD, HC | 29    (18 PD, 11 controls) | PD: 63.7 ± 7.7  HC: 52.4 ± 15.3 | PD: 66.7         HC: 63.6 | GI biopsies (sigmoid colon, rectum, antrum) | ^134^ |
| PD, DLB, MSA, HC | 26    (6 autopsy, 20 live)       Part I: 2 PD, 1 DLB, 1 MSA, 2 controls  Part II: 20 PD, 20 matched controls | PD: 68.8 ± 8.7  HC: 68.0 ± 9.0 | PD/HC: 80 | GI tissue (stomach) | ^100^ |
| PD, HC | 29  (23 PD, 6 HC) | PD: 67  HC: 58 | PD: 40.7  HC: 50.0 | Intestinal mucosa | ^101^ |
| PD, MSA, CBD, PSP | 47     (18 PD, 11 MSA, 6 CBD, 12 PSP) | 64.7 | 53.2 | Olfatory Mucosa (OM) | ^79^ |
| MAS-P, MAS-C, PD, HC | 54  (20 MSA-P, 10 MSA-C, 13 PD, 11 HC) | MSA-P: 60  MSA-C: 61  PD: 63  HC: 42 | MSA-P: 55  MSA-C: 60  PD: 62  HC: 45 | OM | ^78^ |
| iRBD, PD, HC | 163    (63 iRBD, 41 PD, 59 HC) | iRBD: 70  PD: 70  HC:70 | iRBD: 54  PD: 33  HC: 48 | OM  (nasal swab) | ^95^ |
| PD, MSA, iRBD, HC | 342  (107 PD, 99 MSA, 33 iRBD, 103 HC) | PD: 66.6 ± 9.8  MSA: 60.1 ± 7.8  iRBD: 64.4 ± 7.7  HC: 64.0 ± 7.6 | PD: 48.6  MSA: 47.5  iRBD: 30.3  HC: 53.4 | OM | ^127^ |
| PD, MSA, HC | 129   (75 PD, 18 MSA, 36 HC) | PD: 64.1  MSA: 63.3  HC: 59.3 | PD: 34  MSA: 7  HC: 20 | Saliva | ^103^ |
| de novo PD, HC | 67 total    (41 PD, 26 HC) | Not specified | Not specified | Saliva | ^102^ |
|  |  |  |  |  |  |
| PD, LBD, MSA, AD, PSP, CBD, NNC | 130 autopsy abdominal skin   (47 PD, 7 LBD, 3 MSA, 17 AD, 8 PSP, 5 CBD, 43 controls)       30 autopsy scalp skin   (20 PD, 20 non-PD controls) | Autopsy: mean age 76.8        Biopsy: mean age 65.3 | Autopsy: 60.7%  Biopsy: 66% | Skin | ^96^ |
| PD, HC | 64   (34 PD, 30 HC) | PD: 67.1  HC: 58.9 | PD: 70.6  HC: 56.7 | Skin | ^97^ |
| PD, DLB | 69 in vitam   (15 DLB, 13 PD, 41 HC)       49 postmortem   (2 DLB/PD, 7 iLDB, 41 non-LDB) | In vitam: 70  Postmortem: 75 | Not specified | Skin | ^105^ |
| PD, PSP, ET, AD, epilepsy, VE, HC | 617  (332 PD, 28 PSP, 100 Essential Tremor (ET), 32, AD, 42 epilepsy, 6 viral encephalitis (VE), 77 HC) | PD: 65.2  Others (n=285): 60.7 | PD: 60.5  Others: 49.1 | Skin | ^104^ |
| PD, HC | 58   (30 PD, 28 controls with non-α-aSynopathies) | PD: 63.3   HC: 61.1 | PD: 53.3   HC: 2.9 | Skin biopsy extract | ^99^ |
| PD, HC | 80    (30 PD, 50 Controls) | Not specified | Not specified | Neuron-derived extracellular vesicles from blood plasma | ^135,136^ |
| DLB, PD, MSA, CJD, AD, PSP, corticobasal degeneration, IgLON5 tauopathy, FTLD‐TDP, HC | 19 brain samples   (3 DLB, 2 PD, 2 MSA, 4 CJD, 2 AD, 3 PSP, 1 corticobasal degeneration, 1 IgLON5 tauopathy, 1 FTLD‐TDP)  77 CSF  (7 DLB, 1 MSA, 15 LBD/AD, 2 LBD/PART, 3 CJD/LBD, 49 controls) | Not specified | Not specified | CSF  Brain | ^90^ |
| NIH NeuroBioBank  PD, DLB, HC | 6 Brain tissue  (3 PD/DLB, 3 controls)  214 CSF  (88 PD, 58 DLB, 68 controls) | PD: 78.3  DLB: 77.2  HC: 74.3 | PD: 66  DLB: 64  HC: 53 | CSF  Brain | ^86^ |
| AD, DLB, PD, MCI, FTD, HC | 175 CSF and Brain  (119 antemortem + 56 postmortem)  75 AD, 9 DLB, 4 PD, 11 MCI, 13 other dementia, including 10 FTD, 1 mixed dementia, and 2 “other dementia”, 7 cognitively normal controls) | 77.3 | 64.7 | CSF Brain   (frontal cortex, amygdala) | ^71^ |
| From eight available cohorts  DeNoPa, BARMSA, EKUT, KAMSA, UGOT, KIMSA, UPENN, ÑYBB  MSA, PD, DLB, iRBD, HC | DeNoPa (n = 175)  80 PD, 21 iRBD, 60 HC, 14 NS  BARMSA (n = 47)  26 MSA, 2 PD, 19 HC  EKUT (n = 61)  29 MSA, 10 PD, 7 DLB, 15 NS  KAMSA (n = 63)  23 MSA, 5 PD, 3 DLB, 32 NS  UGOT (n = 15)  15 MSA  KIMSA ( n = 36)  18 MSA, 12 PD, 4 HC, 2 NS  UPENN (n = 21)  9 PD, 6 CBD, 1 AGD, 2 LB, 3 GCI  ÑYBB (n = 23)  5 LB, 12 GCI, 5 NS, 1 AD | DeNoPa  PD: 64.5  iRBD: 68  HC: 66.5  Non-aSynopathy: 67  BARMSA  MSA: 65.5  PD: 58.7  HC: 71.9  EKUT  MSA: 61.8  PD: 68.2  DLB: 75.1  Non-aSynopathy: 65.5  KAMSA  MSA: 65.9  PD: 75.4  DLB: 73.9  Non-aSynopathy: 66.7  UGOT  Not specified  KIMSA  MSA: 62.5  PD: 65.5  HC: 64  Non-aSynopathy: 66  UPENN  GCI: 65.3  LB: 48  Non-aSynopathy: 68.8  ÑYBB  Not specified | DeNoPa  PD: 66  IRBD: 62  HC: 65  Non-aSynopathy 64  BARMSA  MSA: 42  PD: 50  HC: 58  EKUT  MSA: 55  PD: 60  DLB: 43  Non-aSynopathy: 40  KAMSA  MSA: 39  PD: 60  DLB: 100  Non-aSynopathy: 50  UGOT  Not specified  KIMSA  MSA: 61  PD: 58  HC: 75  Non-aSynopathy: 0  UPENN  GCI: 67  LB: 67  Non-aSynopathy: 73  ÑYBB  Not specified | CSF  Brain | ^94^ |
| LB, CJD | 269 CSF from NP cohort  (210 LB-, 59 LB+)  604 brain  (CJD) | NP cohort: 69.1 ± 9.8  CJD: 68.4 ± 9.4 | NP cohort: 50.6  CJD: 50 | CSF  Brain | ^70^ |
| Mass General Brigham SCiN (Stem Cells in Neurodegeneration) study  PD, MSA-P, MSA-C, NC | 6 Brain samples  (2 PD, 2 MSA-P, 2 NC)  6 CSF  (2 MSA-C, 2 PD, 2 NC) | Brain: 67.6  CSF: 64.8 | Brain: 50%  CSF: 66.7% | CSF  Brain | ^137^ |
| PD, DLB, MSA, PSP, FTD | 144 CSF   (43 PD, 3 DLB, 31 MSA, 32 PSP, 35 AD)  30 Brain samples  (10 PD, 10 MSA, 4 PSP, 6 controls) | PD: 56.89 ± 10.42  DLB: 59.33 ± 19.14  MSA: 57.26 ± 7.79  PSP: 67.91 ± 7.98  AD: 69.29 ± 7.51  FTD: 67.71 ± 9.30 | PD: 72.1  DLB: 100  MSA: 70.9  PSP: 56.3  AD: 40  FTD: 28.6 | CSF  Brain | ^138^ |
| BOPROPARK cohort (n=61)       PD, iAF, iRBD, Controls | 476   (355 PD, 30 iAF, 19 iRBD, 72 non-neurodegenerative controls) | 65.8 | 63.2 | CSF  Plasma | ^139^ |
| FTD, PSP, CBD, DLB, AD, and SNAP | 316 CSF and plasma + 60 HC plasma  (59 FTD, 31 PSP, 29 CBD, 49 DLB, 97 AD, 51 SNAP, 60 HC) | FTD: 62.9  PSP: 69.2  CBD: 71.3  DLB: 73.7  AD: 67.8  SNAP: 66.2  HC: 61.7 | FTD: 42.4  PSP: 64.5  CBD: 37.9  DLB: 71.4  AD: 44.3  SNAP: 51  HC: 56.7 | CSF  Plasma | ^92^ |
| PD, MSA, DLB, RBD, PSP, AD and PD with PRKN mutations | 470   (221 PD, 39 MSA, 10 DLB, 30 PSP, 25 AD, 17 PD with PRKN mutations, 128 HC) | Serum/CSF: 58.6 | Serum/CSF: 43 | CSF  Serum | ^69,140^ |
| Probable/prodromal DLB, mixed dementia, ND | 81 total    (37 DLB, 6 DLB/AD mixed dementia, 38 non-α-syn NDs including 10 sporadic Creutzfeldt–Jakob disease (sCJD), 10 AD, 8 PSP, 1 CBD, 3 fronto-temporal dementia (FTD)) | Probable DLB: 73.9 ± 6.0    Prodromal DLB: 75.4 ± 4.0    DLB/AD: 66.3 ± 9.6    AD: 69.3 ± 9.6    Probable CJD: 66 ± 16    PSP: 73.5 ± 5.0    CBD: 70   FTD: 72.0 ± 1.6    Other: 53 ± 24.4 | Probable DLB: 71.9    Prodromal DLB: 60    DLB/AD: 50    AD: 70    Probable CJD: 50    PSP: 50    CBD: 0    FTD: 100    Others: 33.3 | CSF  OM | ^91^ |
| IRBD, HC | 132   ( 91 iRBD, 41 Controls) | Not specified | Not specified | CSF  Skin | ^98^ |
| PD, DLB, CBD, HC | 8 CSF  (6 PD, 2 HC)  4 Skin  (2 PD, 1 CBD, 1 HC)  2 OM  (1 PD, 1 CBD)) | CSF: 68.4  Skin: 71.3  OM: 69.5 | CSF: 62.5  Skin: 50  OM: 100 | CSF  Skin  OM | ^26^ |
| PD, non-PD, HC | 422 skin biopsy   (214 PD, 208 non-PD controls including 28 PSP, 30 ET, 35 AD, 42 epilepsy, 6 VE, 66 HC)       20 brain biopsy   (14 PD, 6 HC) | Skin: Not specified  Brain: 58.35 | Skin: Not specified  Brain: 60 | Skin  Brain | ^115^ |
| PD, HC | 124 serum (82 PD, 42 HC)   131 saliva (83 PD, 48 HC) | PD: ~69.2y, HC: ~66.6y | Not specified | Serum  Saliva | ^67^ |
| PD, DLB | 9 brain (4 PD, 5 DLB)  6 skin (PD)  23 intestinal mucosal (PD)  4 CSF (PD) | Brain: ~73.4  Skin: ~81.8  Intestinal mucosal: ~63.7  CSF: ~68.3 | Brain: ~88.8  Skin: ~33.3  Intestinal mucosal: ~43.4  CSF: 50.0 | Brain  Skin  Intestinal mucosal  CSF | ^88^ |
| PD | 44  (20 PD, 18 controls, 6 prion disease) | 71.2±8.7 | CSF/tear fluid: 80 | CSF Tear fluid | ^84^ |
| PD, HC | 1027  (159 HC, 247 NMC, 96 prodromal PD, 525 PD) | HC: 60.6  NMC: 62.0  Prodromal PD: 64.3  PD: 61.5 | HC: 65  NMC: 44  Prodromal PD: 59  PD: 62 | CSF | ^23^ |
| PD | 184  (105 PD, 79 controls) | Controls: 65  PD: 68 | Controls: 37.6  PD: 62.4 | CSF | ^40^ |

AD: Alzheimer’s disease; CBD: Corticobasal degeneration; CI: cognitively impaired; CJD: Creutzfeldt–Jakob disease; CSF: Cerebrospinal fluid; CU: cognitively unimpaired; DLB: Dementia with Lewy bodies; ET: essential tremor; FCx: frontal cortex; FTD: frontotemporal dementia; FTLD-TDP: Frontotemporal Lobar Degeneration with TDP-43 pathology; HC: healthy controls; IDT: indeterminate parkinsonism disease; iAF: idiopathic autonomic failure; iRBD: idiopathic REM sleep behavior disorder; LB: Lewy body; LBD: Lewy body dementia; MCI: mild cognitive impairment; MSA: Multiple System Atrophy; MSA-C: Multiple system atrophy - cerebellar subtype; MSA-P: Multiple system atrophy- parkinsonian type; ND: neurodegenerative disorders; NMC: non-manifesting carriers; NNC: non-neurodegenerative control; PAF: Pure autonomic failure; PD: Parkinson’s disease; PDD: Parkinson’s disease dementia; PSP: progressive supranuclear palsy; SNc: substantia nigra pars compacta; SNAP: Suspected Non-Alzheimer's disease Pathophysiology; VE: viral encephalitis.
